# Takens-Based Kernel Transfer Entropy Connectivity Network for Motor Imagery Classification

**DOI:** 10.3390/s25227067

**Published:** 2025-11-19

**Authors:** Alejandra Gomez-Rivera, Andrés M. Álvarez-Meza, David Cárdenas-Peña, Alvaro Orozco-Gutierrez

**Affiliations:** 1 Signal Processing and Recognition Group, Universidad Nacional de Colombia, Manizales 170003, Colombia; amalvarezme@unal.edu.co; 2 Automatics Research Group, Universidad Tecnológica de Pereira (UTP), Pereira 660003, Colombia; dcardenasp@utp.edu.co (D.C.-P.); aaog@utp.edu.co (A.O.-G.)

**Keywords:** brain–computer interface, electroencephalography, Transfer Entropy, functional connectivity, causal interactions

## Abstract

Reliable decoding of motor imagery (MI) from electroencephalographic signals remains a challenging problem due to their nonlinear, noisy, and non-stationary nature. To address this issue, this work proposes an end-to-end deep learning model, termed TEKTE-Net, that integrates time embeddings with a kernelized Transfer Entropy estimator to infer directed functional connectivity in MI-based brain–computer interface (BCI) systems. The proposed model incorporates a customized convolutional module that performs Takens’ embedding, enabling the decoding of the underlying EEG activity without requiring explicit preprocessing. Further, the architecture estimates nonlinear and time-delayed interactions between cortical regions using Rational Quadratic kernels within a differentiable framework. Evaluation of TEKTE-Net on semi-synthetic causal benchmarks and the BCI Competition IV 2a dataset demonstrates robustness to low signal-to-noise conditions and interpretability through temporal, spatial, and spectral analyses of learned connectivity patterns. In particular, the model automatically highlights contralateral activations during MI and promotes spectral selectivity for the beta and gamma bands. Overall, TEKTE-Net offers a fully trainable estimator of functional brain connectivity for decoding EEG activity, supporting MI-BCI applications, and promoting interpretability of deep learning models.

## 1. Introduction

Brain–computer interfaces (BCIs) establish a direct connection between the human brain and a computer, thereby enabling the capture of neural activity associated with external stimuli or mental tasks without requiring the involvement of peripheral nerves and muscles [1]. The broad potential of BCI has attracted significant attention from applications such as video game control [1], cognitive and emotional state analysis [2], mental disorder diagnosis, and neuromarketing [3]. BCI has been proven helpful in improving concentration and attentional focus [4] and supporting the diagnosis of Attention Deficit Hyperactivity Disorder [5]. Such improvements promoted the integration of BCI technology into educational contexts, resulting in the enhancement of language comprehension in children and older adults [6]. Hence, these interfaces offer opportunities to enhance individuals’ quality of life, contribute to sustainable development by promoting inclusive, equitable, and quality education, and foster lifelong learning opportunities, as recommended by the United Nations [7].

To extend the capabilities of BCI, motor imagery (MI) techniques enable users to control the movement of an agent in either the physical or virtual world by detecting and decoding brain patterns associated with both actual and imagined movements. MI has been the subject of extensive research due to its significant impact on medical applications, including motor assistance, neurorehabilitation, and functional rehabilitation [8]. In fact, MI-based BCIs have proven effective in neuroprosthetic systems, thereby enhancing the quality of life for patients suffering from motor impairments, such as those resulting from spinal cord injuries, amyotrophic lateral sclerosis, or stroke [9]. Additionally, this kind of BCI has demonstrated its efficacy in various clinical applications, such as neurofeedback training therapies for individuals with Parkinson’s disease [10] and the reduction of behavioral manifestations associated with epilepsy [11].

MI-based BCI relies on the analysis of brain activity recorded using either invasive or non-invasive techniques [12]. The former—such as electrocorticography (ECoG) and intracranial electroencephalography (EEG)—offer high spatial resolution, contributing to their effectiveness. However, invasive techniques suffer from surgery risks and gradual degradation of the electrodes. In contrast, non-invasive approaches, including functional magnetic resonance imaging, magnetoencephalography, near-infrared spectroscopy, and superficial EEG, are more commonly used [13]. In particular, EEG signals and MI-based BCI systems suitably match thanks to high temporal resolution, relatively low cost, portability, minimal risks to users, and ease of brain signal acquisition [14]. However, superficial EEG analysis becomes difficult due to the non-stationarity, low signal-to-noise ratio (SNR), and pulsatile movement artifacts [15]. Besides, gaining spatial resolution demands non-user-friendly electrode setups, relegating their use to controlled laboratory conditions [16] and biasing subjects’ posture and mood [1].

Several works have mitigated the above EEG challenges by utilizing traditional machine learning approaches, which include preprocessing, feature extraction, and classification stages. The preprocessing stage aims to overcome issues related to signal quality, easing the identification of BCI-related patterns. The second stage looks for highly interpretable EEG features supporting the signal discrimination, e.g., common spatial patterns [17], its spectral [18] and nonlinear [19] variants, which profit from interchannel relationships. Brain connectivity, a more interpretable set of features, estimates relationships between brain areas or channels, which are either functional or effective. The functional features provide information about temporal correlations between neurophysiological events occurring at spatially distributed locations within the brain network [20]. In contrast, effective features focus on the causal interactions between neuronal units within the same brain network [21]. Despite being widely informative, the independent tuning of features yields suboptimal discriminative machines.

More recent works have applied deep learning (DL) algorithms as a complete BCI processing system, achieving faster and more accurate classifications of sensory input [22]. Nonetheless, conventional DL models lack the interpretability provided by feature engineering, hampering their use in medical and clinical applications that require understanding and explaining machine responses [23]. Advances in the interpretability of DL have enabled the integration of explicit feature extractors into DL architectures through connectivity features such as the phase-locking value [24] or the cross-spectrum [25]. Nonetheless, they are either plugged at the beginning as a precomputation or in the middle as a rigid calculation, reducing the natural flexibility and adaptability of deep models.

This work aims at bridging the gap between connectivity features and deep learning for MI-based BCI applications through a novel end-to-end network for EEG signal classification, termed TEKTE-Net (Takens-based Kernel Transfer Entropy Connectivity Network). Unlike conventional feature-based approaches, TEKTE-Net integrates signal embeddings with a kernelized Transfer Entropy estimator to decode the underlying EEG activity and quantify directed functional connectivity among cortical regions. The architecture employs one-dimensional convolutional layers to highlight spectral discriminative spectral content from each channel, while the Transfer Entropy module models nonlinear and time-delayed pairwise channel interactions within a differentiable learning pipeline. This design enhances both discriminative power and interpretability by linking classification decisions to physiologically meaningful connectivity patterns. The proposed method was validated on a publicly available MI dataset (BCI Competition IV 2a), achieving competitive performance and demonstrating robustness across diverse experimental conditions and inter-subject variability. Overall, the results confirm that TEKTE-Net provides an interpretable, noise-tolerant, and dynamically informed solution for EEG-based motor imagery classification in brain–computer interface (BCI) applications.

The remainder of the work is organized as follows: Section 2 introduces the mathematical framework. Section 3 describes the considered dataset and the experimental setup. Section 4 presents and discusses the results. Finally, Section 5 provides the conclusions and outlines directions for future work.

## 2. Mathematical Framework

This work introduces TEKTE-Net, a novel end-to-end deep learning architecture designed to model nonlinear spatiotemporal dynamics and directed causal interactions directly from raw EEG signals. The model incorporates a customized convolutional module that performs Takens’ embedding, enabling the reconstruction of the underlying dynamical system without requiring explicit preprocessing. This mechanism facilitates the extraction of subject-specific, task-relevant temporal and spectral features. A central contribution of TEKTE-Net is the integration of a fully differentiable Transfer Entropy (TE) estimator, formulated via Rényi’s α-order entropy and positive-definite kernels, allowing for the estimation of nonlinear, directed information flow between EEG channels within a trainable framework. The following sections describe the dataset and mathematical foundations in detail.

### 2.1. Channel-Wise Nonlinear Time Series Embedding from Takens’ Convolutional Layer

Let $X\in R^{T\times C}$ be a discrete multichannel input signal of temporal length *T* and *C* channels. The channel-wise nonlinear filtering of the *c*-th channel is denoted as $\phi_{c}=\varphi \left(x_{c}|\theta_{c}\right)$, being $x_{c}\in R^{T}$ its corresponding input time series, $\phi_{c}\in R^{T_{\phi }}$ the filtered output of length $T_{\phi }\leq T$. A deep learning strategy nonlinearly filters each channel using *L* sequential layers parameterized by $\theta_{c}$, that is, $\varphi \left(\cdot |\theta_{c}\right)=\varphi_{L}(\cdots \left(\varphi_{1}\left(\cdot \right)\right)$. The usual layers considered in the nonlinear filtering are convolutions, poolings, and dropouts that support extracting complex channel-wise temporal structures and nonlinear dependencies to learn more informative and discriminative representations.

Aiming to take advantage of the EEG latent dynamics through the Markovian property, a delay-coordinate Takens’ embedding reconstructs the channel-wise state space by stacking time-delayed versions of the nonlinearly-filtered channel $\phi_{c}$ into fixed-length vectors, resulting in a delay-embedded representation $Z_{c\mu }\in R^{T_{z}\times D}$, where D∈N denotes the embedding order (i.e., number of delay coordinates), the delay offset μ∈N, and $T_{z}\leq T_{\phi }$. A set of fixed, non-trainable convolutional filters implements the embedding replicating delayed copies of the input without altering the learned parameters as

(1) $$Z_{c\mu }=W_{D,\tau ,\mu }^{⊤}*\phi_{c}$$

where the convolution kernel $W_{D,\tau ,\mu }\in {\left\{0,1\right\}}^{R\times D}$, with R=μ+τ·(D−1)+1, depends on three predefined hyperparameters, namely, the delay stride τ∈N, the embedding order *D*, and the delay offset μ. Such Takens’ convolution kernel elements holds elements $w_{r,d}=\delta \left(r-\mu -\tau \cdot d\right)\forall d\in \left[0,D-1\right],r\in \left[0,R-1\right]$, with δ(·) as the discrete delta Dirac function. Thus, the full Takens-embedded nonlinearly-filtered signal $Z_{c\mu }$ enables the subsequent processing layers to access the temporal structure and to capture directed inter-channel interactions through the reconstructed dynamical states rather than raw observations.

### 2.2. Transfer Entropy from Kernel Matrices

This work assesses the inter-channel interactions through the Transfer Entropy (TE) which has been proposed as an effective tool for distinguishing between driving and responding elements, as well as for detecting asymmetries in subsystem interactions [26]. Originally introduced by Schreiber [27] and related to the concept of Granger causality [28], TE operates under the premise that the *c*-th channel $x_{c}$ can be considered to causally influence $c^{\prime }$-th channel $x_{c^{\prime }}$ if incorporating the past of the channel *c*, together with the past of channel $c^{\prime }$, better predicts the present of $x_{c^{\prime }}$ than using $x_{c^{\prime }}$ past alone, for all discrete time instants t∈[1,T]. According to sec:takens, the present of the responding channel $c^{\prime }$ can be computed from the Takens’ delayed-embedding by setting μ=0 and D=1, while its past by setting μ=1 and $D=D^{\prime }$ to be tuned, respectively. In turn, the past of the driving channel *c* accounts for *D* time-delayed interactions, thanks to μ, as in Equation (4).

(2) $$\begin{array}{cc} z_{c^{\prime }0}= & W_{\tau ,1,0}^{⊤}*\phi_{c^{\prime }}\in R^{T_{z}} \end{array}$$

(3) $$\begin{array}{cc} Z_{c^{\prime }1}= & W_{\tau ,D^{\prime },1}^{⊤}*\phi_{c^{\prime }}\in R^{T_{z}\times D^{\prime }} \end{array}$$

(4) $$\begin{array}{cc} Z_{c\mu }= & W_{\tau ,D,\mu }^{⊤}*\phi_{c}\in R^{T_{z}\times D} \end{array}$$

Note that the *t*-th row of the Takens’ embeddings in Equations (2)–(4) correspond to the current state of the responding channel $z_{c^{\prime }t}\in R$, the past state of the responding channel $z_{c^{\prime }t-1}\in R^{D}$, and the past of the driving channel $z_{ct-\mu }\in R^{D}$, respectively, aligned at every time instant $t\in [1,T_{z}]$. Using those three time-aligned embeddings, Equation (5) formally defines the Transfer Entropy $TE(c\to c^{\prime })$ as the expected information gain about the current state of the responding channel given its own past, i.e., $p\left(z_{c^{\prime }t}|z_{c^{\prime }t-1}\right)$, when the past of the driving channel is also known, i.e., $p\left(z_{c^{\prime }t}|z_{c^{\prime }t-1},\;z_{ct-\mu }\right)$.

(5) $$TE\left(c\to c^{\prime }\right)=\;\int \int \int \;p\left(z_{c^{\prime }t},\;z_{c^{\prime }t-1},\;z_{ct-\mu }\right)\;\log\left(\frac{p\left(z_{c^{\prime }}|z_{c^{\prime }t-1},\;z_{ct-\mu }\right)}{p\left(z_{c^{\prime }}|z_{c^{\prime }t-1}\right)}\right)\;dz_{c^{\prime }}\;dz_{c^{\prime }t-1}\;dz_{ct-\mu }.$$

Equivalently, it can be written in expectation form as

(6) $$TE\left(c\to c^{\prime }\right)\;=\;E_{z_{c^{\prime }t},\;z_{c^{\prime }t-1},\;z_{ct-\mu }}\left\{\log\left(\frac{p\left(z_{c^{\prime }t}|z_{c^{\prime }t-1},\;z_{ct-\mu }\right)}{p\left(z_{c^{\prime }t}|z_{c^{\prime }t-1}\right)}\right)\right\},$$

where the expectation operator is defined as

(7) $$E_{z}\left\{f\left(z\right)\right\}\;=\;\int p\left(z\right)\;f\left(z\right)\;dz.$$

Thanks to Bayes’ rule and logarithm properties, the conditional probabilities in Equation (5) are split into four terms depending on the joint and marginals as follows:

(8) $$\begin{array}{cc} TE(c\to c^{\prime }) & =E_{z_{c^{\prime }t},z_{c^{\prime }t-1},z_{ct-\mu }}\left\{\log p\left(z_{c^{\prime }t},z_{c^{\prime }t-1},z_{ct-\mu }\right)\right\}\\ & -E_{z_{c^{\prime }t},z_{c^{\prime }t-1},z_{ct-\mu }}\left\{\log p\left(z_{c^{\prime }t-1},z_{ct-\mu }\right)\right\}\\ & +E_{z_{c^{\prime }t},z_{c^{\prime }t-1},z_{ct-\mu }}\left\{\log p\left(z_{c^{\prime }t-1}\right)\right\}\\ & -E_{z_{c^{\prime }t},z_{c^{\prime }t-1},z_{ct-\mu }}\left\{\log p\left(z_{c^{\prime }t},z_{c^{\prime }t-1}\right)\right\}. \end{array}$$

Since the arguments of the logarithm are marginalizations of $p(z_{c^{\prime }t},z_{c^{\prime }t-1},z_{ct-\mu })$, the expectations are constant on the remaining variables and reduce to

(9) $$\begin{array}{cc} TE(c\to c^{\prime }) & =E_{z_{c^{\prime }t},z_{c^{\prime }t-1},z_{ct-\mu }}\left\{\log p\left(z_{c^{\prime }t},z_{c^{\prime }t-1},z_{ct-\mu }\right)\right\}\\ & -E_{z_{c^{\prime }t-1},z_{ct-\mu }}\left\{\log p\left(z_{c^{\prime }t-1},z_{ct-\mu }\right)\right\}\\ & +E_{z_{c^{\prime }t-1}}\left\{\log p\left(z_{c^{\prime }t-1}\right)\right\}\\ & -E_{z_{c^{\prime }t},z_{c^{\prime }t-1}}\left\{\log p\left(z_{c^{\prime }t},z_{c^{\prime }t-1}\right)\right\}. \end{array}$$

**Figure 1:**
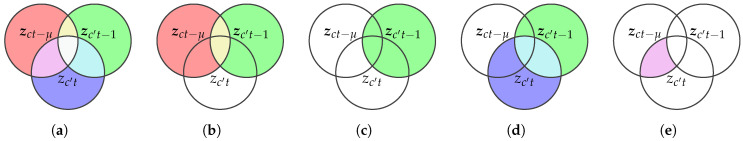
Venn diagrams illustrating the definition of Transfer Entropy from joint and marginal Shannon entropies: Circles correspond to the responding channel present $z_{c^{\prime },t}$ (blue), the Takens-embedded responding’s past $z_{c^{\prime },t-1}$ (green), and the Takens-embedded driving’s past $z_{c,t-\mu }$ (red). The Transfer Entropy identifies the unique predictive information from the driving’s past to the responding’s present beyond the responding’s own history. (**a**) Joint entropy $H_{S}\left\{z_{c^{\prime }t},z_{c^{\prime }t-1},z_{ct-\mu }\right\}$. (**b**) Marginal entropy $H_{S}\left\{z_{c^{\prime }t-1},z_{ct-\mu }\right\}$. (**c**) Marginal entropy $H_{S}\left\{z_{c^{\prime }t-1}\right\}$. (**d**) Marginal entropy $H_{S}\left\{z_{c^{\prime }t},z_{c^{\prime }t-1}\right\}$. (**e**) Resulting $\mathrm{TE}(c\to c^{\prime })$.

Noting that each term in Equation (9) matches the Shannon entropy definition of $H_{S}\left(Z\right)=-E_{z}\left\{\log p\left(z\right)\right\},$ the information gain of the Transfer Entropy is finally rewritten in terms of joint and marginal entropies as in Equation (10). For the sake of visual understanding, Figure 1 illustrates the relationship between the random variables and its entropies in the estimation of the Transfer Entropy.

(10) $$\begin{array}{cc} TE(c\to c^{\prime }) & =-H_{S}\left\{z_{c^{\prime }t},z_{c^{\prime }t-1},z_{ct-\mu }\right\}+H_{S}\left\{z_{c^{\prime }t-1},z_{ct-\mu }\right\}\\ & -H_{S}\left\{z_{c^{\prime }t-1}\right\}+H_{S}\left\{z_{c^{\prime }t},z_{c^{\prime }t-1}\right\}. \end{array}$$

To calculate TE from discrete data, the Shannon entropies in Equation (10) require the estimation of joint and marginal probability distributions, which are often intractable. For avoiding the explicit estimation of these distributions, this work adopts a kernel-based approximation of Shannon joint and marginal entropies from discrete observations over finite state spaces, as defined in Equations (11) and (12) respectively, being v and $v^{\prime }$ are two random vectors [29]. The Gram matrices $K_{v}$ and $K_{v^{\prime }}$ capture the covariance structure of the vector-valued random variables v and $v^{\prime }$ in a Reproducing Kernel Hilbert Space (RKHS), and ∘ denote the Hadamard product operator.

(11) $$\begin{array}{cc} H\left\{v\right\} & \approx S\left(K_{v}\right)=\log\left(\mathrm{trace}\left(K_{v}\right)\right) \end{array}$$

(12) $$\begin{array}{cc} H\left\{v,v^{\prime }\right\} & \approx S\left(\frac{K_{v}\circ K_{v^{\prime }}}{\mathrm{trace}\left(K_{v}\circ K_{v^{\prime }}\right)}\right) \end{array}$$

Above estimators approximate the Transfer Entropy in Equation (10) as

(13) $$\begin{array}{cc} TE_{cc^{\prime }}\approx & -S\left(K_{c^{\prime }0},K_{c^{\prime }1},K_{c\mu }\right)+S\left(K_{c^{\prime }1},K_{c\mu }\right)\\ & -S\left(K_{c^{\prime }1}\right)+S\left(K_{c^{\prime }0},K_{c^{\prime }1}\right), \end{array}$$

(14) $$\begin{array}{cc} K_{c^{\prime }0}= & {\left[\kappa (z_{c^{\prime }t},z_{c^{\prime }s}|\ell_{c^{\prime }0})\right]}_{t,s=1}^{T_{z}} \end{array}$$

(15) $$\begin{array}{cc} K_{c^{\prime }1}= & {\left[\kappa (z_{c^{\prime }t-1},z_{c^{\prime }s-1}|\ell_{c^{\prime }1})\right]}_{t,s=1}^{T_{z}} \end{array}$$

(16) $$\begin{array}{cc} K_{c\mu }= & {\left[\kappa (z_{ct-\mu },z_{cs-\mu }|\ell_{c\mu })\right]}_{t,s=1}^{T_{z}}. \end{array}$$

where the kernel matrices $K_{c^{\prime }0},K_{c^{\prime }1},K_{c,\mu }\in R^{T_{z}\times T_{z}}$ hold the covariances of the responding channel present, its past, and the driving channel past, respectively.

All the three kernel matrices are computed by a positively defined kernel function reproducing a Hilbert space κ(·,·∣ℓ) which holds the parameter set *ℓ*. Particularly, this work proposes to compute the similarities between time series using the Rational Quadratic (RQ) kernel as it generalizes the Gaussian by accommodating multi-scale variation. Equation (17) defines the RQ kernel for a scale mixture parameter α>0 and for the Mahalanobis distance $∥z-z^{\prime }{∥}_{A}^{2}={\left(z-z^{\prime }\right)}^{⊤}AA^{⊤}\left(z-z^{\prime }\right)$ parameterized by the projection matrix A.

(17) $$\kappa \left(z,z^{\prime }\right)={\left(1+\frac{1}{2\alpha }{∥z-z^{\prime }∥}_{A}^{2}\right)}^{-\alpha }$$

Therefore, the kernel parameters in Equations (14)–(16) become $\ell_{c^{\prime }0}=a_{c^{\prime }0}\in R$, $\ell_{c^{\prime }1}\;=\;A_{c^{\prime }1}\in R^{D^{\prime }\times D^{\prime }}$, and $\ell_{c\mu }\;=\;A_{c\mu }\in R^{D\times D}$, and are implemented as dense layers projecting the delay-embedded vectors into new feature spaces tailored to the kernel computation. This interpretation implies that each kernel matrix entry measures distance in an ellipsoidal geometry, where the shape and orientation of the ellipsoids are governed by the trainable matrices $a_{c^{\prime }0},A_{c^{\prime }1},A_{c\mu }$. As a consequence, the dense layers not only learn feature transformations but also define the intrinsic geometry used to compute nonlinear similarity via the Rational Quadratic kernel, while automatically determining the relevance of each embedding axis in the assessment of the TE from *c* to $c^{\prime }$.

### 2.3. Transfer Entropy-Based EEG Classification Model

The above embedded representations and Transfer Entropy estimation hold information about the channel interactions. In the case of EEG signals, such interactions are due to a stimuli, response, or brain state to be identified, hereafter denoted as *y*. Therefore, the nonlinear filtering, Takens’ embedding, Transfer Entropy estimation are gathered into the following single sequential model to be trained for predicting *y*:

(18a) $$\begin{array}{cc} \Phi & =\varphi (X|\theta ) \end{array}$$

(18b) $$\begin{array}{cc} Z & =W*\Phi \end{array}$$

(18c) $$\begin{array}{cc} \mathrm{TE} & =TE(Z|A) \end{array}$$

(18d) $$\begin{array}{cc} p(y|X) & =\sigma (f(\mathrm{vec}(\mathrm{TE}-\mathrm{diag}(\mathrm{TE}))|\vartheta )) \end{array}$$

Firstly, the tensor $\Phi \in R^{T_{\phi }\times C}$ in Equation (18a) holds the nonlinearly-filtered channels as $\Phi ={\left[\varphi \left(x_{c}|\theta_{c}\right)\right]}_{c=1}^{C}$ with parameter set $\theta ={\left\{\theta_{c}\right\}}_{c=1}^{C}$. Secondly, the non-trainable convolution kernel $W=\left[W_{\tau ,1,0}|W_{\tau ,D^{\prime },1}|W_{\tau ,D,\mu }\right]\in R^{R\times (1+D^{\prime }+D)}$ in Equation (18b) stacks the three delayed embeddings in Equations (2)–(4) from each channel into a single tensor $Z={\left[Z_{c}\right]}_{c=1}^{C}\in R^{T_{z}\times \left(D+D^{\prime }+1\right)\times C}$. Then, the function TE(·∣A) in Equation (18c) computes the kernel matrices, and the joint, marginal, and transfer entropies as in Equation (13) with trainable parameter set $A={\left\{a_{c^{\prime }0},A_{c^{\prime }1},A_{c\mu }\right\}}_{cc^{\prime }=1}^{C}$, returning a single C×C matrix $\mathrm{TE}={\left[TE_{cc^{\prime }}\right]}_{c,c^{\prime }=1}^{C}$ holding every pairwise TE from the nonlinearly-filtered Takens’ embeddings. Lastly, Equation (18d) removes the diagonal entries of the TE matrix (representing self-transfers) to avoid informational redundancy, and feeds its flattened version into the ϑ-parameterized fully-connected dense block f(·∣ϑ), followed by σ(·) approximating the posterior class probability. σ(·) corresponds to either the sigmoid (in binary classification) or the softmax function (in multiclass problems).

## 3. Experimental Setup

To address the inherent complexity of brain activity, we propose the TEKTE-Net, a deep model integrating phase space reconstruction through Takens’ embeddings, Transfer Entropy analysis using kernel-based techniques, and an end-to-end trainable architecture. This approach enables the precise extraction of directed connectivity patterns among various brain regions, capturing both dominant and subtle dynamic interactions without constraining the analysis to predefined frequency bands. This section describes the dataset considered to evidence above benefits, which consists on a widely known dataset for EEG-based discrimination of two motor imagery tasks. The section also details the practical considerations for implementing, training, and testing the proposed TEKTE-Net on such dataset.

### 3.1. Dataset and Preprocessing

**Figure 2:**
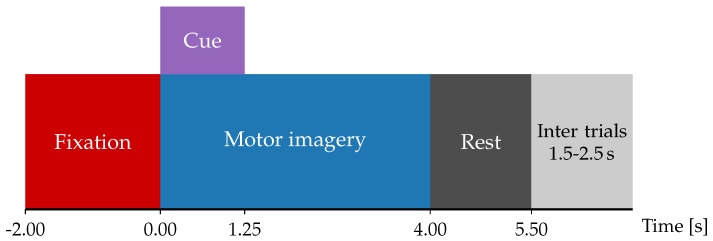
Schematic description of the Motor Imagery (MI) paradigm.

To validate our TE-based EEG classification approach, this work considers the BCI Competition IV dataset 2a (BCICIV2a), a publicly available resource composed of EEG recordings from nine healthy participants. These recordings were obtained during multiple trials of a motor imagery (MI) experiment with the protocol illustrated in Figure 2. In each trial, participants first see a fixation cross on a computer screen, accompanied by an auditory cue. Two seconds later, a directional arrow appears for 1.25 s, signaling the start (t=0) of one of four possible MI tasks: left-hand, right-hand, both feet, or tongue movement. One second after the MI signal, subjects begin performing the instructed task during three seconds (i.e., until the 4-s mark after the instruction), at which point the cross disappears. A brief 1.5-s pause follows, during which the screen remains blank. For validation purposes, this study excludes feet and tongue movement tasks and focuses solely on binary classification of trials instructed to move the left (y=0) and right (y=1) hands. For each subject, the BCI Competition IV provides two subsets with the same experimental protocol: the training subset holds between 113 and 138 trials per subject, and the testing subset holds between 108 and 142 per subject [30].

**Figure 3:**
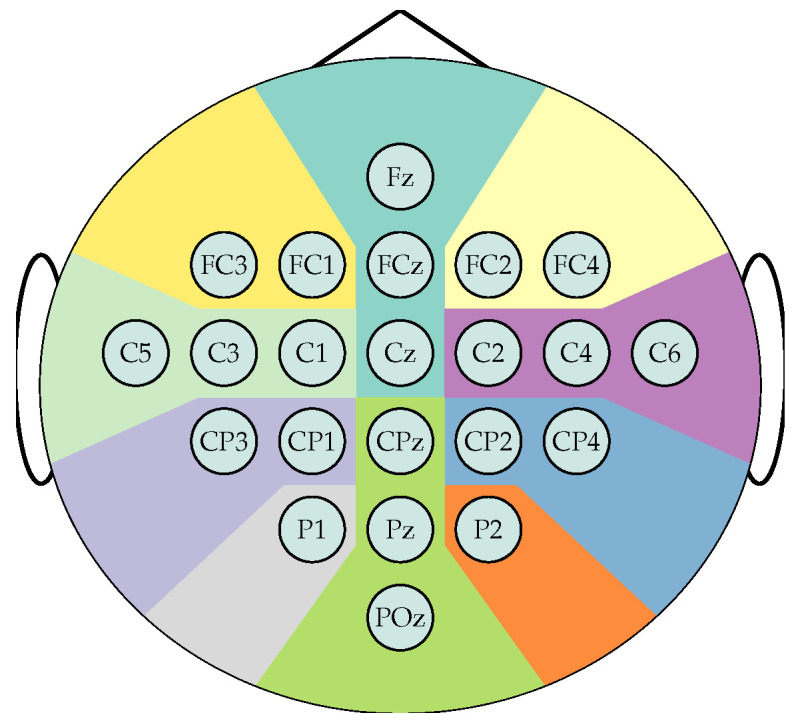
Sensor positions according the 10-20 electrode placement system for the BCI Competition IV 2a dataset. 22 channels are distributed over ten main brain regions: 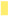 Frontal left, 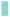 Frontal, 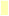 Frontal right, 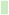 Central left, 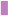 Central right, 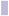 Centro-parietal left, 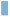 Centro-parietal right, 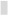 Parietal left, 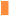 Parietal right, 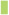 Posterior.

The EEG data were recorded using C=22 Ag/AgCl electrodes, placed according to the international 10/20 system as shown in Figure 3. The data preprocessing applies a 50 Hz notch filter and a bandpass filter between 0.5 and 100 Hz and resamples to 250 Hz. Therefore, each participant comprises a subset of 22 channels and *N* samples, and classification is based solely on labels 1 and 2, which distinguish between right-hand and left-hand motor imagery, respectively. Trials are split into two-seconds windows with 0.5 s overlap, that is T=500, from which the segments from 2.0 to 4.0 s after cue onset are selected to train the model as they are expected to hold the most relevant information to the MI task. Lastly, the spherical splines method applies the surface Laplacian transform to each segment to reduce the influence of low spatial frequency activity, minimize the occurrence of spurious channel connectivities, and mitigate the volume conduction effect [31,32,33,34].

### 3.2. Semi-Synthetic Causal EEG Benchmark

**Figure 4:**
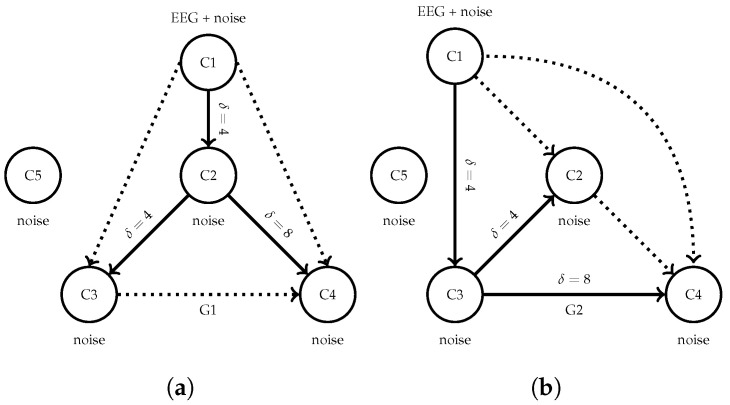
Causal structures of the synthetic benchmarks used for validation. Each configuration consists of one real EEG, three synthetic, and one noise channels. Causal interactions are different bewteen the two structures. (**a**) G1. (**b**) G2.

The evaluation of TEKTE-Net’s ability to infer directed functional connectivity from EEG signals relies on a causality classification benchmark with semi-synthetic data. Two simulated connectivity models generated the surrogate data holding different causality flow interactions [35,36]. Similar to the work of Kus et al. [37], the two causal configurations, hereinafter referred to as G1 and G2, were designed by introducing directional interactions among five virtual EEG channels. Figure 4 illustrates both configurations, evidencing different dominant causal flows while shared parameters for the time-delayed interactions and a common channel with Gaussian noise. Unlike a straightforward simulated scenario, the channel C1 in both generators was directly initialized with EEG recordings from the BCICIV2a dataset, ensuring semi-synthetic trials that preserve realistic, non-stationary neural dynamics. Each model configuration generated the remaining four channels according to the predefined causal interactions, plus Gaussian noise with power varying from −6 dB to +3 dB with respect to the base EEG variance. Sampling random channels from BCICIV2a and running each model yielded a surrogate supervised EEG dataset with 1000 trials labeled according to their interaction structure (500 from G1 and 500 from G2).

### 3.3. Model Setup and Hyperparameter Tuning

The proposed TEKTE-Net follows a sequential deep learning architecture, implemented as a three-block end-to-end model, as detailed in Table 1.

The first block applies the nonlinear Takens’ embedding described in Section 2.1, beginning with a channel-wise convolution of *C K*-length trainable convolution kernels, $\theta ={\left\{w_{c}\in R^{K}\right\}}_{c=1}^{C}$, which the model then feeds into an average pooling layer for temporal aggregation and noise robustness. Non-trainable embedding matrices split each channel in the pooling output as responding present, responding past, and driving past signals for the estimation of Transfer Entropy. The second block estimates the pairwise TE by computing the kernel matrix for each nonlinear Takens’ embedding parameterized by three trainable projection matrices. The last block acts as a nonlinear classifier, with complexity controlled by the number of hidden units *H*, where TE connectivities serve as input features and $\hat{y}$ represents the estimated posterior for *O* classes. This work considers only one output unit due to the binary classification tasks at hand (G1 vs. G2 in the semi-synthetic benchmark and left vs. right in the BCICIV2a dataset). Therefore, the implemented model depends on five hyperparameters to be tuned ($K,D^{\prime },D$, τ, and μ), and three sets of trainable parameters: the convolutional filters, the projection matrices in the RQ kernel, and the weighting matrices in the head block.

To mitigate inter-subject variability, a Bayesian optimization stage identified the hyperparameters of the model in Table 1 that maximize validation accuracy on a participant-specific basis. The search space for the key hyperparameters controlling the model architecture included the convolutional kernel size K∈[3,5,…,125], the time stride τ∈[1,5], the time delay for the interaction μ∈[0,10], and the embedding order for the responding and the driving channels $D,D^{\prime }\in \left[1,10\right]$. During the search, the optimizer filtered out hyperparameter configurations with size issues due to the kernel size, pooling size, and embedding dimensions. Each valid configuration was trained for a maximum of 1000 epochs with a batch size of 32, using the binary cross-entropy loss function and the Adam optimizer. An early stopping mechanism, monitoring of the validation loss with a patience of 5 epochs, prevented overfitting and reduced unnecessary training time.

Regarding Bayesian optimization, the following setup controlled the search behavior and efficiency: Up to ten search trials and a kernel regularization of $10^{-4}$ for promoting numerical stability and robustness to noisy objective evaluations, a trade-off of 2.6 for a moderate balance between exploration and exploitation, and a stratified 5-fold cross-validation data splitting for mitigating model overfitting during the search. Along with the Bayesian optimization setup, the resulting hyperparameters aim not only to achieve better performance metrics but also to relate the subject’s performance to its optimal architecture, thereby improving the model’s interpretability.

Performance metrics, including accuracy, sensitivity (true positive rate), specificity (true negative rate), and F1-score, assess the quality of the resulting optimal hyperparameters on both the training and testing subsets in terms of. The fold-wise computation of metrics enables the reporting of mean and standard deviation statistics subject-dependently, for a better understanding of the model’s performance.

## 4. Results and Discussion

### 4.1. Performance on Semi-Synthetic Causal EEG Data

**Figure 5:**
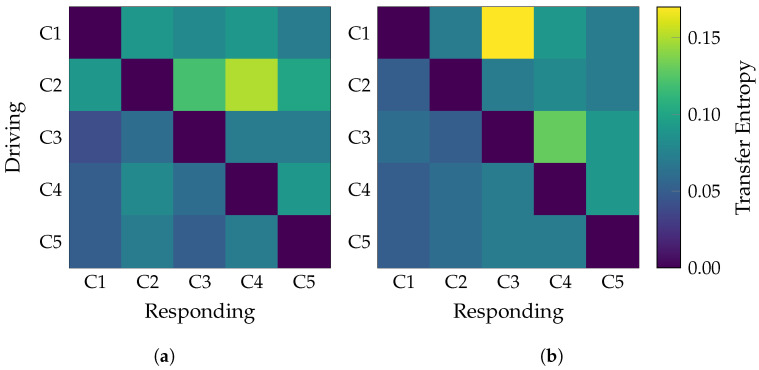
Average TE matrices for the semi-synthetic causal structures G1 and G2. Each matrix captures the dominant directed interactions estimated by the TEKTE-Net. (**a**) G1-Transfer Entropy Matrix. (**b**) G2-Transfer Entropy Matrix.

For the semi-synthetic dataset, the tuning procedure in sec:hyperparamsetup yielded the following configuration: $D=8,D^{\prime }=3,\tau =2,\mu =4,K=95$. Such a configuration achieved the performance metrics on the five-fold cross-validation: Accuracy = 97.0±1.7, Sensitivity = 96.8±2.2, Specificity = 97.4±1.5, F1-score = 97.0±1.7. The average class-wise TE matrices in Figure 5 illustrate the effectiveness of TEKTE-Net in decoding the simulated interactions. For either configuration, TEKTE-Net highlighted the dominant connections (C1→C2, C2→C3, and C2→C4 in G1; and C1→C3, C3→C4 in G2), validating the model’s ability to infer directed causal relationships even in the presence of Gaussian noise and temporal delays, consistent with previous studies on TE applied to neural signals [27,35,38]. However, the C3→C2 pathway, intended to be a key causal link in G2, was not clearly detected, suggesting a possible limitation of the model in capturing weak or highly noise-sensitive pathways. Interestingly, unexpected connections C2→C1 in G1 and C1→C4 in G2 emerged. Indirect interactions can explain the former through non-causal statistical dependencies or implicit feedback effects induced by the model’s nonlinearity and the mixture of real EEG signals (in C1) with structured noise in C2. The latter, likely mediated by C3, evidences the model’s sensitivity to multi-step interactions. Previous works discuss such effects on directed connectivity, where the asymmetry of measures like TE can still reflect spurious correlations or undesired symmetries [39,40]. Lastly, low TE values appeared flowing to C5, even though it only holds noise. These weak activations may arise from residual statistical dependencies or shared variance leakage, phenomena frequently reported in the literature on directed connectivity using information-theoretic metrics and observed in other approaches for estimating causal interactions from synthetic data [41].

**Figure 6:**
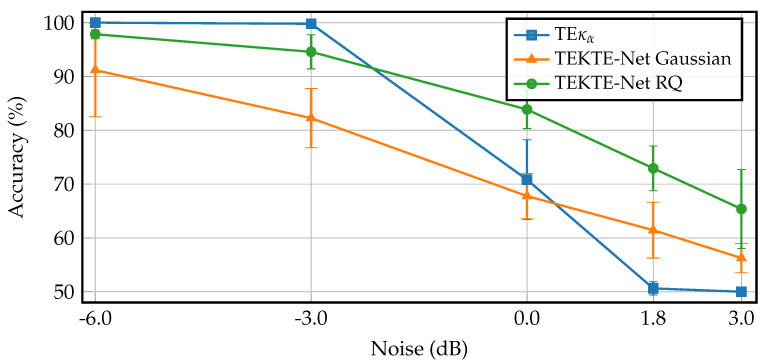
Classification accuracy of the TE$\kappa_{\alpha }$, Gaussian, and Quadratic kernel models under increasing Gaussian noise levels. Noise is expressed in decibels (dB), where lower values correspond to cleaner signals. The Quadratic model shows the most stable performance across the noise range.

For the sake of a noise robustness study, Figure 6 compares the proposed TEKTE-Net with the RQ kernel against the $TE\kappa_{\alpha }$ connectivity, another kernel-based Transfer Entropy estimator, ref. [42] and the TEKTE-Net with a Gaussian kernel at varying noise levels. Firstly, note that the baseline $TE\kappa_{\alpha }$ model achieved perfect classification accuracy under low-noise conditions (−6.0 dB). However, its performance sharply degrades beyond 0 dB, reaching nearly 50% at the highest noise level (+3.0 dB). The TEKTE-Net with a Gaussian kernel, while more robust than $TE\kappa_{\alpha }$, consistently underperformed the RQ kernel. A closer look reveals that the TEKTE-Net with the RQ kernel reduced classification accuracy by 32.5%, followed by the Gaussian kernel with a 35.0% reduction, and $TE\kappa_{\alpha }$ with the most severe performance drop of 50.0%. Such results confirm the superior robustness of the proposed model under noisy conditions, thanks to the designed end-to-end training framework, in contrast to $TE\kappa_{\alpha }$, which lacks a learning scheme beyond the classification stage.

**Figure 7:**
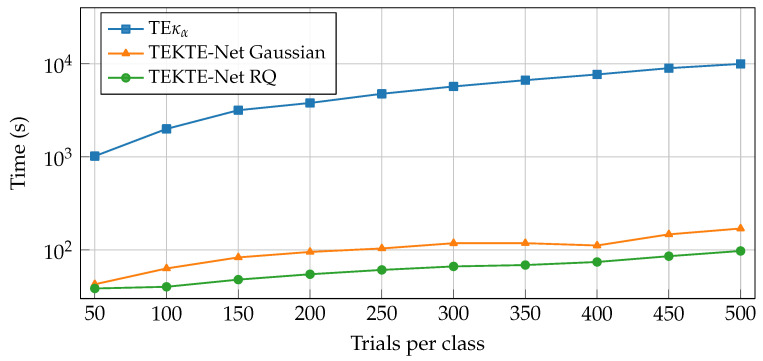
Scalability test for $TE\kappa_{\alpha }$ and TEKTE-Net: Total training vs. the number of trials per class.

Subsequently, to assess the computational scalability of the models, a comparison was performed between the $TE_{\kappa_{\alpha }}$ method and TEKTE-Net with both Gaussian and rational quadratic (RQ) kernels, considering the total training time as a function of the number of trials per class. For an even comparison, the scalability experiments run on identical hardware and software, utilizing an NVIDIA Quadro RTX 5000 GPU equipped with eight processing cores, one socket, and eight physical cores with 16 threads, along with 16 GB of RAM. Previous setup ensures that the observed differences in training time are solely attributable to the computational complexity of the models, rather than variations in the execution environment. As shown in Figure 7, the training time of the $TE_{\kappa_{\alpha }}$ method increases exponentially, reaching nearly $10^{4}$ s for 500 trials per class. This behavior stems from its reliance on non-parametric kernel estimations and operations with quadratic complexity relative to the number of samples, which severely restricts its scalability in large-data scenarios. In contrast, TEKTE-Net, with either a Gaussian or RQ kernel, substantially improves scalability, maintaining total training times within the order of $10^{2}$ s. However, although the architectural backbone remains identical, the Gaussian kernel requires a greater number of kernel evaluations and more computationally expensive exponential operations, resulting in slightly higher training times compared to the RQ kernel. The rational quadratic kernel, which introduces an additional scale mixture term, achieves a more flexible representation while preserving computational efficiency. Consequently, the superior scalability and efficient parallelization capabilities of TEKTE-Net RQ on the GPU lead to its adoption in the final model implementation and subsequent experiments.

### 4.2. Hyperparameter Tuning

**Figure 8:**
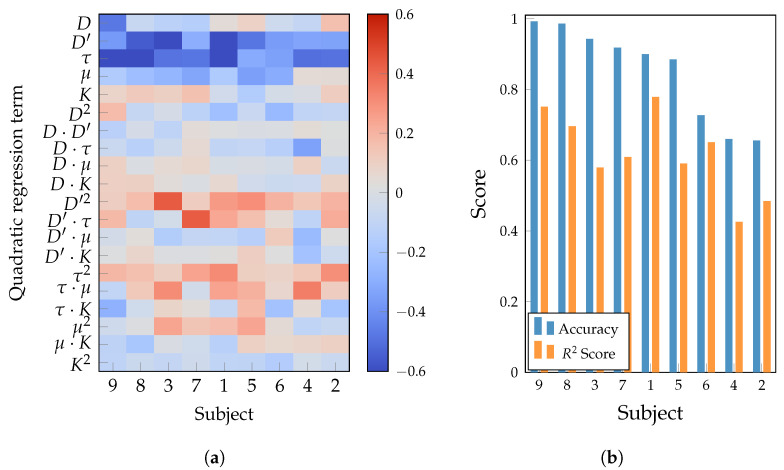
Results of the Bayesian hyperparameter optimization for subjects descending-sorted according their validation accuracy. (**a**) Quadratic regression coefficients from validation accuracy to hyperparameters. (**b**) Regression and classification performances for validation data.

For a deeper understanding of the influence of hyperparameters on performance, a quadratic regression analysis was conducted on the configurations explored through the Bayesian optimization described in Section 3.3. This approach provides a structured view of the nonlinear effects and cross-interactions within the hyperparameter–performance landscape. Figure 8a illustrates the standardized regression coefficients (linear terms, second-order terms, and pairwise interactions) for each subject. At a global level, the coefficient map from the quadratic regression analysis reveals three robust regularities. (i) The temporal stride τ and the embedding order for the responding channel $D^{\prime }$ exhibit a predominantly negative linear effect across subjects (blue bands), indicating that increasing both hyperparameters without compensation tends to reduce accuracy. (ii) The positive coefficients of the quadratic and interaction terms involving τ, notably $\tau^{2}$, τ·K, and τ·μ, indicate nonlinear dependencies, suggesting that coordinated adjustments of the kernel size *K* and the interaction delay μ can partially compensate for shifts in the stride. (iii) The embedding dimension $D^{\prime }$ mainly contribute through subject-specific curvature or interactions (e.g., $D^{\prime 2}$, $D^{\prime }\cdot \tau$). These regularities align with previous works, which suggest that models are more tunable in subjects exhibiting structured response surfaces [43,44].

Figure 8b contrasts each subject’s validation accuracy with the $R^{2}$ of the quadratic surrogate fitted to the hyperparameter response surface. In general, higher accuracies co-occur with larger $R^{2}$, suggesting a more structured and informative optimization landscape: In the high-performing group, accuracy and $R^{2}$ decline in tandem, indicating coherent curvature and stable hyperparameter interactions that TEKTE-Net can exploit. The mid-performing group follows the same tendency with two nuances: Subjects 7 and 5 yield moderate $R^{2}$ values consistent with their accuracies; whereas subject 1 attains a comparatively high $R^{2}$ despite mid-range accuracy, implying that the surrogate captures meaningful curvature while subject-specific factors may constrain performance (e.g., residual noise or ceiling effects). In addition, the low accuracies at generally smaller $R^{2}$ in the low-performing group suggest weaker or less discriminative neural signatures and reduced SNR. In such cases, Bayesian optimization faces a more diffuse, noisy landscape and yields limited gains. This interpretation aligns with reports of poor signal quality and potential BCI illiteracy [45,46]. Finally, Table 2 lists the per-subject optimal hyperparameters and stratifies subjects according to validation accuracy.

**Figure 9:**
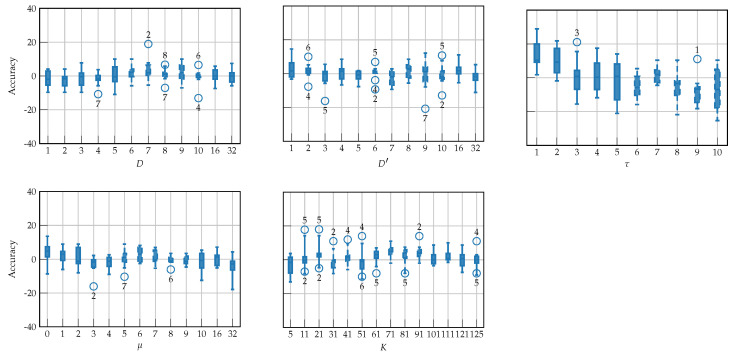
One-at-a-time (OAT) sensitivity analysis for the TEKTE-Net hyperparameters. Boxplots illustrate the distribution of deviations in validation accuracy when varying a single hyperparameter around its subject-wise optimum.

To validate the model’s sensitivity to changes in hyperparameters, a perturbation experiment is conducted using a one-at-a-time (OAT) analysis, where a single hyperparameter is varied across a predefined range while keeping all others fixed at their optimal values, shown in Table 2. This experiment captures the local influence of each hyperparameter on validation accuracy, yielding interpretable OAT curves that characterize model robustness near the optimal architecture. Boxplots in Figure 9 summarize the accuracy gain or loss with respect to the optimal architecture as a function of each hyperparameter across subjects. The model exhibits a relatively robust performance, with most hyperparameters (*D*, $D^{\prime }$, μ, and *K*) producing fluctuations within approximately ±10% in validation accuracy. This stability suggests that the model is inherently regularized and exhibits low sensitivity to moderate hyperparameter perturbations, reflecting a smooth optimization landscape where performance remains consistent across a wide range of configurations. In contrast, the stride parameter τ shows the highest sensitivity, as seen by the wider spread and performance degradation trend in its boxplot. Since the stride controls the frequency-domain information captured by the temporal embeddings, its pronounced sensitivity indicates that frequency resolution and phase alignment are essential for optimizing discriminative performance. Lastly, the outliers visible across all hyperparameters primarily correspond to the low-performing group, corroborating that poor signal quality, reduced discriminative neural patterns, and BCI illiteracy result in greater performance variability and less structured optimization surfaces [45,46].

### 4.3. Interpretability Analysis

For assessing model interpretability, a subject-specific analysis was conducted across temporal, spatial, and spectral axes. The temporal analysis employs a sliding-window approach for understanding time engagement and training/testing variations. On the spatial axis, the activated TE aims at interpreting functional connectivity patterns. The spectral analysis of channel-wise learned filters focuses on understanding frequency specialization. All analyses were performed under the optimized hyperparameters summarized in Table 2.

**Figure 10:**
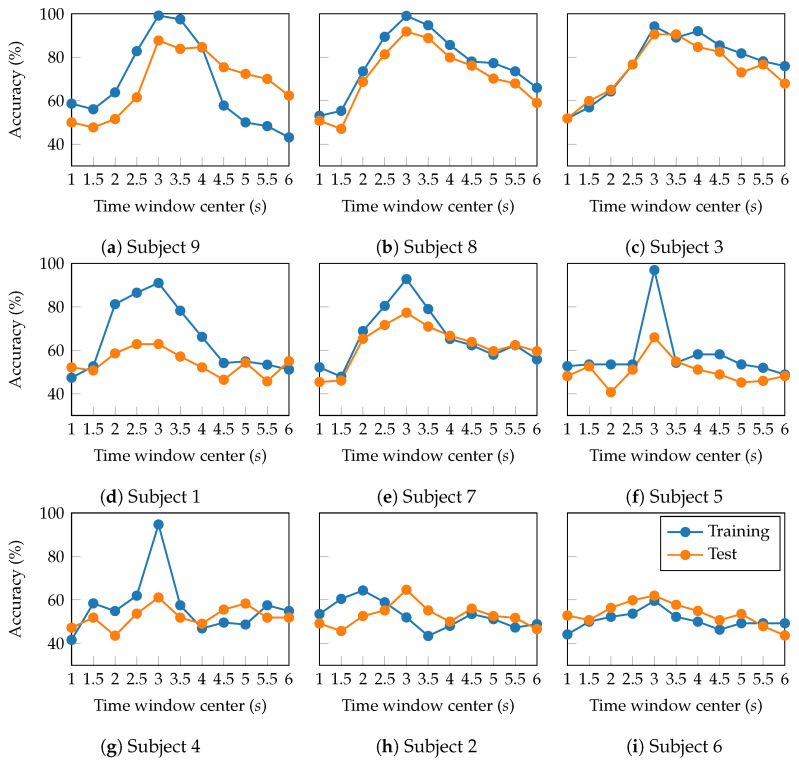
Classification accuracy for each subject across overlapping time windows during the 7-s motor imagery task. A total of 11 time windows were used, each with a 0.5-s overlap. The x-axis represents the center of each time window, and the y-axis shows the classification accuracy (%). This analysis reveals temporal dynamics in classification performance throughout the motor imagery period.

Sliding window analysis enables the exploration of MI engagement variability by examining the temporal evolution of classification accuracy. Figure 10 presents the attained accuracy as a two-second window is slid along the trials (from 0 to 7 s), with subjects sorted according to their performance in Figure 8b. At first glance, consistent peak classification accuracies appear within the time window between 2 and 4 s after the task onset. Previous studies have demonstrated that this window range suitably captures the most discriminative MI-related patterns by effectively reflecting the evolution of sensorimotor rhythms during the active phase of cognitive processing [47].

Furthermore, the temporal performance profiles cluster subjects into three groups. In the group of high-performing subjects (Subjects 9, 8, and 3), TEKTE-Net exhibited a marked and consistent increase in accuracy of over 90% within the 2–4 s window. Despite the slight decline towards the trial end, the proposed model maintained relatively high test accuracy levels (above 60%), suggesting the presence of stable and task-relevant neural activity in these subjects. On subjects 7, 1, and 5 (mid-performing), the proposed model performed moderately, with test accuracy peaking between 60% and 80% within the 2- and 4-s windows. The temporal profile of this group exhibits a clear training-to-test accuracy gap, followed by a gradual decline in training accuracy. Such a profile indicates existing MI-related neural patterns, but these are less consistent and more susceptible to cognitive variability than those in the high-performing group [48]. For the group of low-performing subjects (Subjects 6, 4, and 2), the accuracy evolved flatly or with high variability around the chance level (between 45% and 60%). Particularly, Subject 6 presented severe overfitting characterized by an abrupt difference between training and test subsets in the window centered at three seconds. Such an accuracy gap is a symptom of inconsistent or weak MI-related neural patterns, which limit the reliability of classification. Therefore, the temporal performance profiles revealed differences in the quality and steadiness of the neural patterns among subjects, proving the need for subject-dependent MI models.

For spatial interpretability, the well-established activation maximization technique optimizes the model’s output for each motor imagery class with respect to the TE layer. This interpretability technique enables the identification of TEKTE-NET connectivities that strongly contribute to class-specific activations. It also allows for the analysis of the spatial distribution and intensity of interactions between brain regions, providing a clear depiction of the functional dynamics underlying motor imagery tasks.

**Figure 11:**
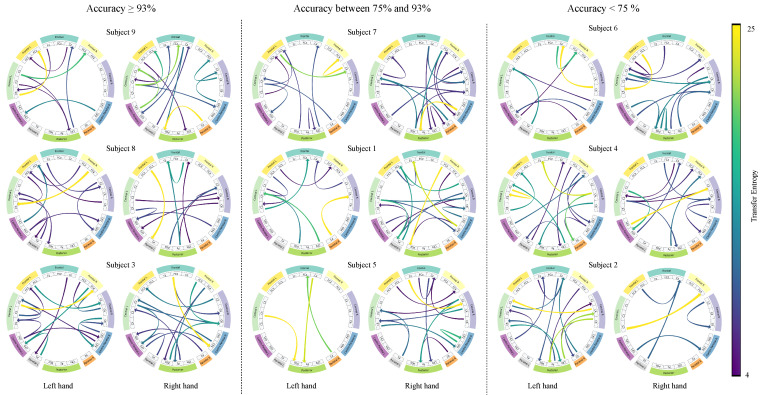
Brain connectivity patterns during left- and right-hand motor imagery tasks across models grouped by test accuracy. High performed (accuracy ≥ 93%), mid performed (accuracy between 75% and 93%), and low performed (accuracy < 75%) display distinct activation distributions. Each row corresponds to a representative model within each performed group.

Figure 11 highlights the directionality, magnitude, and causal nature of the 5% largest interactions between cortical regions, computed under the Transfer Entropy framework, across the nine subjects grouped according to their performance in Figure 8b. In the high-performing group, causal activation patterns exhibited contralateral connections involving frontal, central, and parietal areas, emphasizing the directionality and magnitude of these connections. For instance, Subject 9 revealed strong paths including C5→FC1, C3→FCz, and P2→POz, while Subject 8 highlighted the FC4→C5 and FC1→P1 connections for left and right hand MI, respectively. In the mid-performing subjects, causal interactions are more widespread and less contralateralized than in the previous group, hence partially aligning with MI-related patterns. Remarkably, connections CP4→Fz and FC2→POz, in Subject 1, C3→POz and Cz→Pz, in Subject 5, suggest compensatory recruitment of posterior regions. Conversely, the activations in the low-performing group lack contralateral organization, with atypical interhemispheric causal connections. For instance, the connections between the left-frontal and left-central channels in Subject 6 are atypical in the MI task [49]. Additionally, the right hemisphere is responsible for most of the highlighted connections in Subjects 2 and 4. Therefore, the spatial and causal interpretation of TEKTE-Net elucidates the relationship between functional connectivity and motor imagery decoding performance, demonstrating efficient sensorimotor integration for the task at hand in the first group [50,51] and the compensatory recruitment of posterior regions in the second group [52].

**Figure 12:**
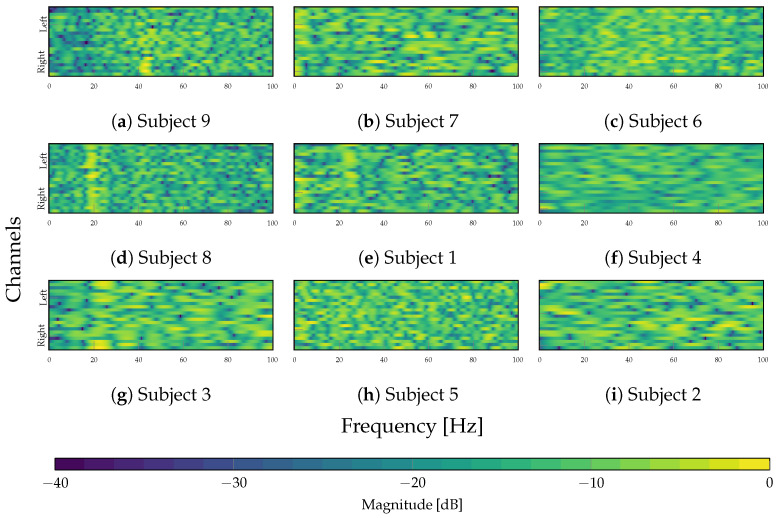
Frequency responses (dB) of the filters learned by the depthwise layer. In each panel, the *x*-axis shows frequency (Hz) and the *y*-axis shows the 22 EEG channels; the color indicates the average magnitude in dB.

The characterization of the spectral behavior of the TEKTE-Net relies on the frequency response of the channel-wise learned filters for each subject. The frequency response results from the fold-averaged magnitude of the Fast Fourier Transform computed for each convolution kernel. Figure 12 presents the resulting magnitude response (in dB) along the frequency (0 to 100 Hz) and the channels (from left to right), thereby visually assessing variability among the subject groups and the selectivity of the learned filters across the frequency spectrum.

According to Figure 12, the proposed model yields a more structured spectral pattern in the high-performing subjects (first column) than in the rest of the cohort. In particular, the filters exhibited a band-pass-like structure in Subjects 3 and 8 around 20 Hz, with a slight rightward predominance in Subject 8. From a neurophysiological perspective, the beta band (20 Hz) is associated with sensorimotor coordination and exhibits contralateral lateralization to movement, particularly within the left hemisphere during motor or spatial-orienting tasks [53]. In the case of Subject 9, the filters displayed an unattenuated response around the gamma band (40 Hz), primarily concentrated in the right hemisphere, frequently linked to sustained attention, perceptual integration, and executive control processes [54]. Hence, the structured frequency response suggests that the depthwise convolutional filters adapt to preserve the oscillatory components, relevant for the classification task, effectively acting as spectral selectors that maintain frequency regions containing discriminative neural information.

Regarding the second group and third groups (center and right columns), the spectral behavior was less defined compared to the first group. Only the learned filters for Subject 1 partially selected frequencies around 25 Hz over the left hemisphere, similar to the best-performed subjects, albeit with lower clarity and spatial extent. For Subjects 7 and 5, the filters develop a spurious magnitude response, lacking spectral or spatial alignment. Contrarily, the depthwise convolutional filters for the latter group yield an elongated and flat spectral response, reflecting the absence of frequency selectivity. Therefore, the TEKTE-Net’s spectral specialization positively relates to classification performance, as the low power of MI-related rhythms, particularly beta and gamma, is associated with lower cognitive performance and reduced classification accuracy [55,56].

### 4.4. Performance Assessment

This work evaluates TEKTE-Net’s performance on the official testing set from the BCI Competition IV Dataset 2a. Table 3 presents the per-subject performance metrics on this subset, sorted by validation accuracy. In general, note that the testing accuracy naturally decreased by approximately 7% compared to the validation one, due to the intrinsic within-subject variability. However, the average F1 score of over 80% indicates that TEKTE-Net successfully identified individual motor imagery patterns, demonstrating a reasonable generalization capability. For the high-performing group, the model consistently reached scores of over 90%. Such a result indicates that TEKTE-Net finds well-structured neurophysiological patterns with both precision and generalizability. For the mid-performing group, TEKTE-Net yields F1 scores between 75% and 85%, followed by the most significant accuracy drops compared to the validation set. Such a finding indicates transitional signal regimes or moderate generalization characteristics, due to accentuated between-session variability. The low-performing group remains just above the chance levels, suggesting highly intrinsic neurophysiological noise or BCI illiteracy. It is also worth mentioning that Subjects 5, 4, and 2 present the most difference between Sensitivity and Specificity, with F1 scores over 65%, despite the class balance provided by the competition. Hence, the heterogeneity in class distributions emerges as the most reasonable explanation for uneven metrics on such subjects, which facilitates the labeling of the positive class while hindering the negative. Therefore, the stratification and subject-wise analysis highlight the necessity of adaptive or personalized strategies in BCI decoding frameworks, while providing a deeper understanding of the inner workings of TEKTE-Net across individuals.

**Figure 13:**
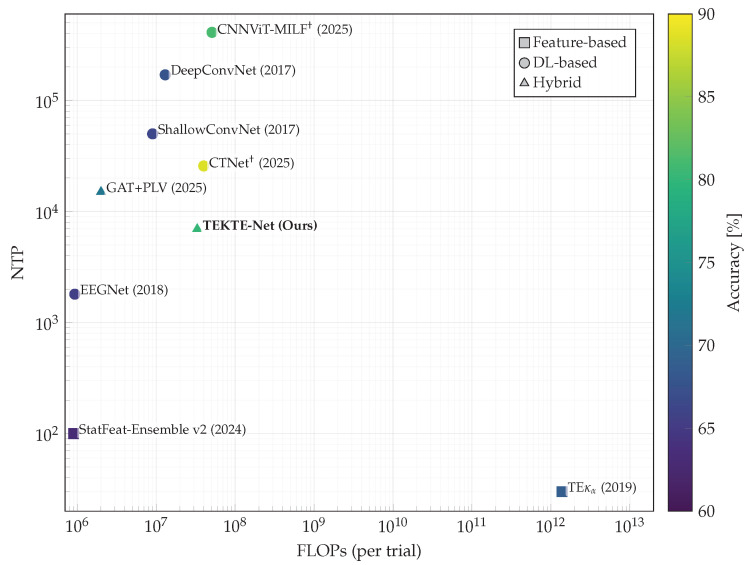
State-of-the-art comparison for binary MI-EEG decoding in terms of Floating Point Operations (FLOPs), number of trainable parameters (NTP), and testing accuracy. _†_ Accuracy reported on the BCI Competition IV-2b testing set (Left vs. Right).

Figure 13 contrasts the proposed TEKTE-Net against state-of-the-art approaches on the testing sets from the BCI Competition IV in terms of Floating Point Operations (FLOPs), number of trainable parameters (NTP), and average accuracy (color-coded). Both, FLOPs and NTP, describe the computational efficiency and complexity of models, which directly impact performance, scalability, and deployability. Contrasted approaches range from traditional feature engineering techniques (square mark), through deep learning architectures (circle mark), to hybrid models (triangle mark). The formers include time-frequency, dynamic nonlinear [57,58], and entropy-based connectivity features [42], which achieved accuracies ranging from 60% to 70%. While these methods benefit from high interpretability, they rely heavily on handcrafted statistical features and traditional classifiers. This reliance limits their ability to model the complex spatiotemporal characteristics inherent to EEG signals, which in turn constrains their generalization capacity and scalability to diverse experimental conditions. The seminal deep learning models, explicitly designed for EEG decoding [59,60], introduce automated feature extraction pipelines that reach accuracies up to 68%. Hence, the between-session variability hampers their performance due to overfitting and a lack of inherent regularization through parameterized features. More recently, Transformer-based architectures have shown competitiveness in capturing long-range dependencies through self-attention and a global receptive field, facilitating the integration of multi-channel information. For instance, CTNet reports 88.49±9.03% on BCI IV-2b (two classes) [61], while CNNViT attains 81.11% on BCI IV-2b under a subject-independent LOSOCV protocol [62]. However, both models demand larger computational and memory resources, substantial amounts of data (or pretraining), careful hyperparameter tuning, and offer more limited neurophysiological interpretability. In turn, the TEKTE-Net and GAT+PLV proposals introduce hybrid frameworks that integrate functional connectivity measures with deep learning architectures. In particular, GAT+PLV feeds a Graph Attention Network with Phase-Locking Value connectivity [24]. On the official test set, TEKTE-Net achieves an accuracy of 80.12%, surpassing GAT+PLV by 8.21%, which requires the explicit precomputation of phase coupling (PLV). These findings suggest that connectivity-informed, end-to-end architectures outperform traditional deep networks and hybrid architectures, while being competitive against modern transformer-based models.

## 5. Concluding Remarks and Future Work

This work introduces TEKTE-Net, an end-to-end deep learning model integrating directed EEG functional connectivity for EEG discrimination. TEKTE-Net develops an information-theoretic kernelization of Transfer Entropy within a channel-wise convolutional architecture, enabling the joint modeling of nonlinear delayed and directional neural dependencies. The proposed approach combines five key components: Firstly, a channel-wise convolutional block specializes spectral filters to keep discriminative frequency components. Secondly, a kernelized Transfer Entropy captures time-delayed signal interactions while preserving causal structure. Third, a Bayesian optimization strategy tunes the architectural hyperparameters to maximize decoding accuracy and to support model interpretability. Fourth, a quadratic kernel formulation enhances robustness to low signal-to-noise ratios, a key characteristic of real-world EEG data. Finally, unlike conventional feature engineering or other hybrid approaches, TEKTE-Net operates in a fully differentiable and end-to-end fashion, without requiring handcrafted features, explicit causality priors, or manual preprocessing steps.

Experimental results across both semi-synthetic causal benchmarks and a real MI-EEG dataset demonstrated that TEKTE-Net accurately recovered the intended causal structures and achieved competitive decoding performance. On semi-synthetic EEG data, the model successfully inferred predefined directed interactions, achieving an average validation accuracy above 97%. The resulting Transfer Entropy matrices reproduced the imposed topologies even under Gaussian noise, confirming the framework’s ability to identify true directed interactions. The noise analysis revealed that the quadratic kernel variant degrades more slowly as noise levels increase, validating its robustness under varying SNR.

The temporal, spatial, and spectral analyses provided critical insights into both decoding dynamics and underlying neurophysiological processes. Sliding-window experiments confirmed that the 2–4 s post-cue interval consistently yielded the highest decoding performance, aligning with known sensorimotor activation phases during MI. Connectivity visualizations demonstrated clear distinctions between high, medium, and low performing: high-performing subjects exhibited contralaterally organized, sensorimotor-coherent networks, whereas low-performing users showed diffuse, vertically distributed connections lacking lateralization. Spectral analysis of the learned filters further revealed that filters for high-performing subjects preserved power in the β (≈20 Hz) and γ (≈40 Hz) bands, associated with motor coordination and cognitive engagement, respectively. Conversely, widespread attenuation in low performing reflected diminished task-related activity. These findings provide a direct neurophysiological rationale for the observed decoding variability, validates the extraction of neurophysiologically meaningful patterns, and demonstrates the model’s interpretability at the network level.

From an implementation standpoint, TEKTE-Net maintains a modular and interpretable structure that allows seamless integration into existing BCI pipelines. Its information-theoretic regularization improves robustness to noise, while the kernelized architecture ensures flexibility across subjects and recording conditions. Moreover, the model’s differentiable formulation enables direct gradient-based optimization, fostering explainability and traceability—two features often absent in black-box EEG decoders. Nevertheless, estimating pairwise Transfer Entropy (TE) across channels using kernel matrices introduces a computational cost that depends on the number of channels and the lag configuration. Such a cost grows faster for TEKTE-Net than for standard CNNs. In practice, channel selection strategies may reduce this overhead without degrading performance, thereby facilitating real-time deployment or operation in resource-constrained settings [63].

The proposed future work identifies three key research directions: First, we will pursue attention-guided alignment of directed interactions together with dynamic kernel adaptation to improve cross-subject generalization while preserving interpretability. Second, we will integrate TEKTE-Net within a unified BCI framework, considering active, reactive, and passive paradigms to guide online adaptation and system integration. In particular, we will examine how interpretable directed-connectivity patterns can function as real-time markers of cognitive or attentional states in reactive/passive BCIs for monitoring engagement and mental workload [64]. Third, we will test whether subject-specific connectivity profiles from TEKTE-Net can serve as neurophysiological biomarkers for clinical applications, such as tracking motor recovery after stroke and assessing motor-imagery performance in cohorts with neurodegenerative diseases or psychosis. Such a test will validate the framework’s translational value for clinical neuroengineering and personalized rehabilitation [65,66].

## Figures and Tables

**Table 1:** Detailed TEKTE-Net architecture for MI classification.

Layer	Variable	Dimension	Hyperparameters
Input	X	C×T×1	–
DepthwiseConv1D		C×(T−K+1)×1	Kernel size *K* Stride = 1 ReLU activation
AveragePooling1D	Φ	$C\times T^{\prime }\times 1$	Pool size = 4 Stride = 4
TakensConv1D	$z_{c^{\prime }0}$	$C\times T_{z}\times 1$	–
	$Z_{c^{\prime }1}$	$C\times T_{z}\times D^{\prime }$	Order $D^{\prime }$
	$Z_{c\mu }$	$C\times T_{z}\times D$	Order *D* Stride τ Delayed interaction μ
RationalQuadratic Kernel	$K_{c^{\prime }0}$ $K_{c^{\prime }1}$ $K_{c\mu }$	$C\times T_{z}\times T_{z}$ $C\times T_{z}\times T_{z}$ $C\times T_{z}\times T_{z}$	Scale mixture rate α=1
TransferEntropy	TE	C×C	–
Flatten		C×(C−1)	–
Dense		*H*	Hidden units H=10 ReLU activation
Dense	$\hat{y}$	*O*	Output units O=1 Sigmoid activation

**Table 2:** TEKTE-Net hyperparameters tuned through the Bayesian optimization sorted by descending validation accuracy.

Group	Subject	*D*	$D^{\prime }$	τ	μ	*K*
High	9	3	2	1	5	125
	8	1	3	1	3	123
	3	3	8	2	0	63
Mid	7	5	1	1	3	81
	1	1	1	1	8	91
	5	6	6	5	9	121
Low	6	5	5	4	10	99
	4	4	3	2	8	51
	2	10	2	1	0	57

**Table 3:** Performance metrics achieved by TEKTE-Net on the validation and official testing sets. Subjects are sorted in descending order of validation accuracy. Mean and standard deviation for each metric are computed from a five-fold cross-validation.

Subject	Val. Acc (%)	Acc. (%)	F1 (%)	Sens. (%)	Spec. (%)
9	99.1±1.9	90.0	90.1	90.8	89.2
8	98.5±2.0	92.5	92.8	94.1	90.9
3	94.2±6.0	93.4	93.9	98.6	88.1
7	91.7±6.9	77.1	78.7	85.5	69.0
1	89.9±8.3	85.1	85.3	87.1	83.1
5	88.4±5.1	74.8	75.0	78.5	71.4
6	72.6±8.4	73.1	71.3	65.5	81.1
4	72.3±13.7	75.0	74.8	75.4	74.6
2	67.7±8.4	59.9	66.7	80.3	39.4
Avg	86.0±2.5	80.1	81.0	84.0	76.3

## Data Availability

The dataset 2a from the BCI Competition IV, used in the validation of the current study, is publicly available at https://bbci.de/competition/iv/download/ (accessed on 1 June 2025).
